# Complete Genome Sequences of Allofrancisella inopinata SYSU YG23 and Allofrancisella frigidaquae SYSU 10HL1970, Isolated from Water from Cooling Systems in China

**DOI:** 10.1128/MRA.00554-20

**Published:** 2020-11-25

**Authors:** Caroline Öhrman, Ingrid Uneklint, Linda Karlsson, Daniel Svensson, Mats Forsman, Andreas Sjödin

**Affiliations:** a Swedish Defence Research Agency, CBRN Defence and Security, Umeå, Sweden; University of Maryland School of Medicine

## Abstract

Near neighbors to the causative agent of tularemia, Francisella tularensis, isolated from diverse sources, have been reported in recent years. In this announcement, we present the complete sequences of circular chromosomes of one of the closest neighboring genera of *Francisella* (i.e., the type strains of Allofrancisella inopinata and Allofrancisella frigidaquae).

## Abstract (precis)

Near neighbors to the causative agent of tularemia, Francisella tularensis, isolated from diverse sources, have been reported in recent years. In this announcement, we present the complete sequence of the circular chromosome of one of the closest neighboring genera of *Francisella*, the type strains of Allofrancisella inopinata and Allofrancisella frigidaquae.

## ANNOUNCEMENT

Recently, a new genus within the family *Francisellaceae*, named *Allofrancisella*, was published, with three different species (1, 2). We present the complete sequences of the chromosome of the type strain of Allofrancisella inopinata SYSU YG23 (DSM 101834, FSC1302) and of the type strain of Allofrancisella frigidaquae SYSU 10HL1970 (DSM 101835, FSC1303), in addition to the already published complete genome sequence of the type strain of Allofrancisella guangzhouensis (3), previously named *Francisella guangzhouensis* (4). The strains of this genus are all isolated from water collected from different cooling systems in the city of Guangzhou, China (4, 5). Both strains were obtained from the DSMZ (German Collection of Microorganisms and Cell Cultures; DSM 101834 and DSM 101835). The strains were grown on McLeod agar supplemented with bovine hemoglobin (Becton, Dickinson, San Jose, CA) and IsoVitalex (provided by Umeå University Hospital) at room temperature and with 5% CO_2_ for 24 to 48 h, and DNA was extracted with a magnetic bead-based protocol (MagAttract high-molecular-weight DNA kit; Qiagen, Hilden, Germany). Short-read sequencing libraries were prepared using a Nextera XT 600-cycle kit (Illumina, San Diego, CA) and sequenced on an Illumina MiSeq sequencer, followed by trimming using Trimmomatic (6) and quality checking with FastQC v0.11.9 (7) prior to assembly. The long-read sequencing library, with no shearing or size selection, was prepared using ligation sequencing kit 1D (SQK-LSK108), barcoded with native barcoding kit 1D (EXP-NBD103), and sequenced on a Nanopore MinION flow cell, FLO-MIN106 (R9.4). All steps were performed according to the manufacturer’s instructions unless stated otherwise. Nanopore reads were basecalled using Albacore v1.1.1 (Oxford Nanopore Technologies, Oxford, UK) and trimmed with Porechop (8) prior to assembly. See Table 1 for the read statistics. Using both Illumina and Nanopore reads as the input, complete circular chromosomes were generated using Unicycler 0.4.7 (9), with the setting --startgenes, with complementary *dnaA* sequences for available *Francisella* species as the input. As reported by Unicycler, both assemblies were made with five rounds of pilon, and FSC1302 was rotated to the start of *dnaA*. No ambiguities were found when comparing the final hybrid assemblies using DNAdiff of the MUMmer package (10), with a short-read assembly, generated with an abyss-pe 2.2.2 k value of 51 (11). Nanopore reads were mapped to the hybrid assembly using minimap2 (12), and by viewing the bam files in Integrative Genomics Viewer (13), circularization could be confirmed by >10× coverage. Two structural variations were reported using Sniffles (14), a 339-bp inversion at nucleotide position 1520923 and an 81-bp insertion located in a repeat area at nucleotide position 1646356. Both variations were corrected to generate the final assembly. The corrections were verified by more than 10× coverage by both Illumina and Nanopore reads using the same mapping procedure as that described above. The annotation of the final assembly was performed using NCBI Prokaryotic Genome Annotation Pipeline (15, 16). See Table 1 for genome statistics. All software programs were executed using default settings unless otherwise stated.

**TABLE 1:** Read and genome statistics

Isolate	Bacterial species	Strain ID	DSM no.	No. of MiSeq reads	MiSeq mean read length (bp)	No. of MinION reads	MinION *N*_50_ (bp)	Genome size (bp)	GC%	No. of protein coding sequences	GenBank accession no.	SRA accession no.
SYSU YG23	Allofrancisella inopinata	FSC1302	101834	491,152	142.5	71,483	14,144	1,750,491	32.0	1,534	CP038241	SRR11853071, SRR11853072
SYSU 10HL1970	Allofrancisella frigidaquae	FSC1303	101835	852,168	143.7	149,804	9,351	1,674,180	32.1	1,480	CP038017	SRR11853073, SRR11853074

### Data availability.

These complete genome sequences are the first versions and have been deposited in GenBank/SRA. Genome and read sequences for SYSU YG23 (FSC1302) are available under the accession no. CP038241, SRR11853071, and SRR11853072 and for SYSU 10HL1970 (FSC1303) under the accession no. CP038017, SRR11853073, and SRR11853074.
